# Tired Nerves, Neurasthenia

**Published:** 1915-02

**Authors:** 


**Tired Nerves, Neurasthenia.** Dr. J. A. Cotton, "Iowa Med. Jour," Des Moines, Iowa, in a lengthy paper on Neurasthenia says, "without the nervous system there would be no aches, no pains; but without a nervous system there would be no life, for today it is well understood the nervous system controls and operates every organ, every function of the human body, and if normal action is to be forthcoming this system must be kept as near par as possible." The writer goes thoroughly into the subject of a specific nerve food, saying the great majority of nervous conditions, in fact a goodly number of systemic ailments, where a true pathological cause cannot be found, is due to a want of nerve nutrition.

Much stress is laid on the fact nerve nutrition consists of lecithin, nuclein and phosphorus but the latter must be given in its elementary form, and not as a compound; if otherwise, phosphorus is not obtained. He speaks most highly of the phosphatometer, saying since its advent it is now not necessary to guess as to whether sedatives or tonics are necessary but decides the case positively in a very few minutes.

Several cases of neurasthenia are reported, in which after various methods of treatment have entirely failed, the Index showed a nerve-cell hunger, which was supplied by phosphorus (the Compound Phosphorus Mixture (Dowd) was used) and the happiest results followed.

